# Inhibitory Effect of Sihuangxiechai Decoction on Ovalbumin-Induced Airway Inflammation in Guinea Pigs

**DOI:** 10.1155/2014/965429

**Published:** 2014-07-02

**Authors:** Xue Ping Huang, En Xue Tao, Zhan Qin Feng, Zhao Lu Yang, Wei Fen Zhang

**Affiliations:** ^1^Department of Radiology, Affiliated Hospital of Weifang Medical University, No. 2428, Yuhe Street, Weifang, Shandong 261031, China; ^2^Universtiy Hospital of Weifang Medical University, Weifang, Shandong 261053, China; ^3^College of Pharmacy and Biological Science, Weifang Medical University, No. 7166, Baotong West Street, Weifang, Shandong 261053, China

## Abstract

The aim of this study was to investigate the effect of sihuangxiechai decoction on asthmatic Guinea pig model which was sensitized by intraperitoneal (i.p.) injection of ovalbumin (OVA) and challenged by OVA inhalation to induce chronic airway inflammation. Differential cell counts of cytospins were performed after staining with Giemsa solution. The quantity of leukocytes and its classification in bronchoalveolar lavage fluid (BALF) and blood were evaluated by blood cell analyzer and microscope. Histological analysis of the lung was performed by hematoxylin and eosin (H&E) staining. The levels of interleukin-4 (IL-4) and tumor necrosis factor-alpha (TNF-*α*) in BALF and serum were detected by radioimmunoassay (RIA). The total number of leukocytes in BALF and blood has no significant difference between Sihuangxiechaitang decoction treated group and dexamethasone (DXM) treated group but was significantly lower than those of asthma group. The percentage of eosinophils in lung tissues of sihuangxiechai decoction treated group was significantly lower than that of asthma group. The results demonstrated that the levels of IL-4 and TNF-*α* in the sihuangxiechai decoction treated group were significantly reduced compared with the asthma group. In conclusion, these findings demonstrate that sihuangxiechai decoction has a protective effect on OVA-induced asthma in reducing airway inflammation and airway hyperresponsiveness (AHR) in a Guinea pig model and may be useful as an adjuvant therapy for the treatment of bronchial asthma.

## 1. Introduction

Bronchial asthma is the most common chronic respiratory disease which is seriously damaging to people's health. The prevalence of asthma is markedly increasing worldwide, and it has been included to be a significant cause of morbidity and mortality in developed countries [1, 2]. It has been broadly recognized that asthma is characterized by structural alterations in the airways [3] and variable degrees of chronic inflammation which can lead to recurrent episodes of wheezing, breathlessness, chest tightness, and cough. This chronic disease also causes bronchospasm, bronchial mucosal thickening from edema, eosinophilic infiltration, bronchial wall remodeling, and excessive mucus production and can ultimately lead to airway obstruction [4–6]. These reactions have been referred to as airway remodeling, which is considered to occur as a result of an imbalance in the mechanism of airway regeneration and repair. Recent studies indicate that asthma is a chronic inflammatory airway disease that is caused by a variety of cells like eosinophils (Eos), mast cells, neutrophils, T lymphocytes, airway epithelial cells, and a number of cytokines [7]. These cells secrete several chemical mediators, such as major basic protein (MBP), eosinophil cationic protein (ECP), eosinophil peroxidase (EPO), lipid ecosystems, elastase, and Th2 cytokines, such as IL-4 (a switch factor for IgE synthesis), IL-5, and IL-13 [8]. Therefore, these cells are considered as major targets for basic and therapeutic research. Among them, Eos together with Th2 cytokines IL-4, IL-5, and IL-13 may ultimately contribute to AHR in asthma; it can also cause airway inflammation in the initial and effector phase stages of allergy [9, 10]. Toxic proteins produced and released by Eos could injure airway epithelium directly; then, remodeling is associated with more severe airflow obstruction; therefore, AHR is produced. There is evidence that Eos inflammation of the airway is involved in the risk of exacerbations. Eos has important antigen presenting function. It also could be used as antigen presenting cell (APC) that participates in the pathogenesis of asthma by producing the potent cytokine IL-4. Meanwhile, IL-4 can also promote Th0 differentiation into Th2 and produce a large number of cytokines. Many characteristics of asthma are deemed to reflect consequences of Th2 cell-dominated immune responses to allergens. Furthermore, allergen-specific Th2 cells play a pivotal role in the pathogenesis of asthma. Airway Eos, together with IL-4, IL-5, and IL-13, may directly act on epithelial and smooth muscle cells in airway epithelium to induce mucus hyperproduction, goblet cell hyperplasia, and AHR [7, 11–13]. In addition, IgE plays a crucial role in the propagation of airway inflammation in allergic asthma. It is well known that IgE levels positively correlate with the presence of asthma symptoms, probability for allergic sensitization [14].

Likewise, tumor necrosis factor-alpha (TNF-*α*) is considered to be a significant proinflammation and the promoter of the airway inflammation in asthma. Both IL-13 and TNF-*α* are well known as remodeling associated cytokines [15]. Furthermore, asthma inflammation is also induced by cytokines released from TNF-*α*, which can increase the production of more inflammatory mediators, including IL-8, granulocyte-macrophage colony-stimulating factor (GM-CSF), and other cytokines. Clinical observation and animal experimentation indicated that the content of TNF-*α* in BALF was elevated when asthma attacks [16–19].

Since the establishment of the doctrine of airway inflammation, anti-inflammatory therapy had an irreplaceable role in asthma. Because of its anti-inflammatory, antiallergy, and other pharmacological effects, glucocorticoids have become the first-line drugs for treating asthma [20]. Although corticosteroids can significantly ameliorate airway inflammation and inhibit Eos infiltration and airway inflammatory mediator release, it cannot change the course of the disease. A lot of practice has proved that traditional Chinese medicine (TCM) has the holism in mind and superiority in synthetic action in the treatment of asthma. In previous report, the compounds of Chinese medicines (*Astragalus membranaceus*, honey-fried herba ephedrae, stir-baked radix scutellariae, rhizoma polygonati, scorpio, centipede, radix bupleuri, and cassia twig) were considered to improve the patient's constitution, regulate immune function, and enhance the anti-inflammatory and antiallergic ability and it has enjoyed satisfied clinical application [21]. For example,* Astragalus membranaceus* (Huangqi) are amongst the most popular health-promoting herbs in China; their use dates back to more than 2000 years and were recorded in* Shen Nong's Materia Medica* written in the Han Dynasty. It has also been used in the treatment of diabetes mellitus, nephritis, leukemia, and uterine cancer [22]. In addition,* Scutellaria baicalensis* is a widely used Chinese herbal medicine that has been used historically in anti-inflammatory and anticancer therapy [23].

In this paper, we investigated the effect of sihuangxiechai decoction applied to allergic diseases. The ovalbumin (OVA) was used to induce asthma Guinea pig model which evaluated the possible effects and mechanisms of sihuangxiechai decoction on inflammation and systemic immune responses. According to the research of quantitative analysis of inflammation and the role of cytokines in OVA-induced asthma Guinea pig model, the aim of this study was to explore the mechanism of Chinese medicine prescriptions on asthma and mine new drugs which could enhance immunity, improve the body's defenses function, enhance the resistance to disease, and provide pharmacodynamics and mechanism of the experimental basis for clinical.

## 2. Materials and Methods

### 2.1. Animal

32 healthy male Guinea pigs (weighing 250 ± 30 g) were purchased from Lukang & Co. (Jining, Shandong, China). The Guinea pigs were housed under standard laboratory conditions for one week. All Guinea pigs were provided with food and tap water* ad libitum*. All experimental procedures were carried out in accordance with the NIH Guidelines for the Care and Use of Laboratory Animals, and animal handling followed the dictates of the National Animal Welfare Law of China [24, 25].

### 2.2. Materials

Ovalbumin (OVA) was obtained from Sigma Company (Sigma & Co., American) and purchased from Beijing Branch Company after packing. Dexamethasone was purchased from Yongning Pharmaceutical Co., Ltd. (Jinan, Shandong, China), batch number: 0203131. Sihuangxiechai decoction was provided by the pharmacy in our hospital. The radioimmunoassay (RIA) kits of IL-4 and TNF-*α* were purchased from Science and Technology Development Center of PLA General Hospital. Piston compressor nebulizer was manufactured by San Up S.A. (San Up S.A., Argentina). *γ*-counter was produced by Shanghai annular company.

### 2.3. Preparation of Sihuangxiechai Decoction

This formula is a dried decoction of a mixture of 16 medicinal herbs (Table 1). The quality of each crude drug was tested in accordance with the pharmacopoeia of China. The herbs were soaked in cold water, which were dissolved in the 2 times bigger volume of water, boiled for 30 min, filtered, and boiled with slow fire for 20 min, poured out the supernatant fluid about 200 mL. Then the dregs were added in boiling water in the same way with 3 replications. All the liquid was combined and concentrated to 2 g/mL. Moreover, scorpion and centipede were ground into powder and added into the liquid directly.

### 2.4. Experimental Group and Drug Treatment

**Figure 1:**
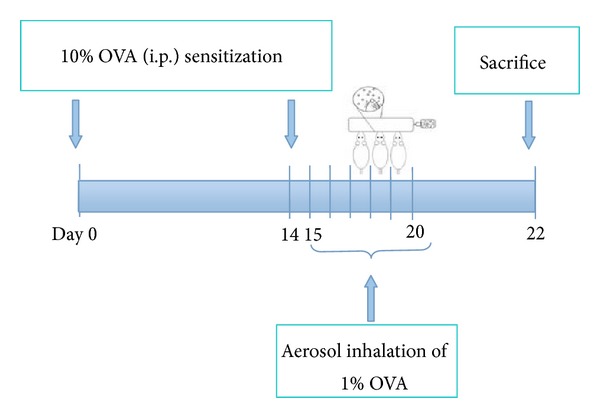
Schematic representation of the experiment procedure. The Guinea pigs were sensitized with intraperitoneal (i.p.) injection of OVA on day 0 and challenged with aerosolized OVA for six consecutive days 14 days later. The animals were sacrificed 24 hours after the last challenge, and tissue samples were collected on day 22.

The experimental groups (*n* = 8) were randomly divided into four groups as follows: normal group, asthma group, dexamethasone-treated group (DXM group), and traditional Chinese medicine group (TCM group). Except normal group, the remaining groups of animals were induced by intraperitoneal injection of 1 mL of 10% OVA on day 0. After 14 days, the oversensitized animals showed asthma symptoms while inhaling with an aerosol of 20 min of 1% OVA using piston compressor nebulizer for 6 days every other day (Figure 1). The OVA-sensitized animals were treated with TCM (10 g/kg/day) suspended in NS (0.9%) by oral gavage 30 minutes before the OVA challenge. Normal group and asthma group were treated with only NS (0.9%, 10 g/kg/day) by oral gavage, and the positive control group (DXM group) was treated with DXM (2 mg/kg/day) by intraperitoneal injection (i.p.) before the OVA challenge [26].

### 2.5. Bronchoalveolar Lavage Fluid (BALF)

24 h after the last OVA challenge, all Guinea pigs were anesthetized by intraperitoneal injection of pentobarbital (50 mg/kg, 3%). Thoracotomy was performed according to standard surgical procedures. BALF was collected by flushing 1 mL of NS (0.9%) into the lung via the trachea immediately after sacrifice. Approximately 0.8 mL of BALF was recovered after three lavages. The BALF was centrifuged (400 g, 4°C, 5 min) and the supernatant was stored at −70°C until measurement of cytokines. Cells from the BALF were washed three times with PBS and the pellet was resuspended in 100 *μ*L of phosphate buffer solution (PBS). Total cell number was counted and differential cell counts of cytospins were performed after staining with Giemsa solution. The cells were differentiated by general leukocyte morphology and 200 cells were counted in each of four random locations.

### 2.6. Blood Collection

After BALF was collected, the Guinea pigs were euthanized by aortic exsanguination. The artery blood was collected in different tubes with sodium citrate anticoagulant or gel procoagulant for different tests and centrifuged at 4°C (4000 rpm) for 10 min [27]. Subsequently, the serum was stored at −70°C for measurement of cytokines [20].

### 2.7. Measurement of IL-4 and TNF-*α* Using Radioimmunoassay (RIA)

IL-4 and TNF-*α* concentrations of each sample were detected by radioimmunoassay (RIA) according to the manufacturer's instructions. Samples and standard products were mixed with IL-4 and TNF-*α* antibodies and then incubated at 37°C for 20 minutes. After centrifugation (3500 r/min, 4°C), the supernatant in each tube was measured by *γ*-counts. Finally, according to the standard curve, the concentrations of IL-4 and TNF-*α* were calculated. All samples and standards were assayed in duplicate.

### 2.8. Histological Analysis

Prior to the removal of the lung, the lung tissue and trachea were filled intratracheally with fixative (10% formaldehyde) using a ligature around the trachea. Lung tissue was fixed in 10% formaldehyde. The tissues were dehydrated in various concentrations of ethanol and embedded in paraffin. For histopathological examination, 4 *μ*m sections fixed tissues were cut on a microtome (Leica RM 2235, Microsystems Nussloch GmbH, Germany), placed on glass slides, deparaffinized, and stained with H&E for general morphology. Tissue lesion and inflammatory cell infiltration were then examined using microscope [27–29].

### 2.9. Statistical Analysis

All of the quantitative data analyses were performed using statistical software programs SPSS 17.0. All results were expressed as means ± standard errors of the mean. All statistical significance of differences was assessed by one-way ANOVA followed by Student's *t*-tests. In all cases, *P* < 0.05 was considered statistically significant.

## 3. Result

### 3.1. Effects of TCM on OVA-Induced Total and Differential Leukocytes in BALF

**Figure 2:**
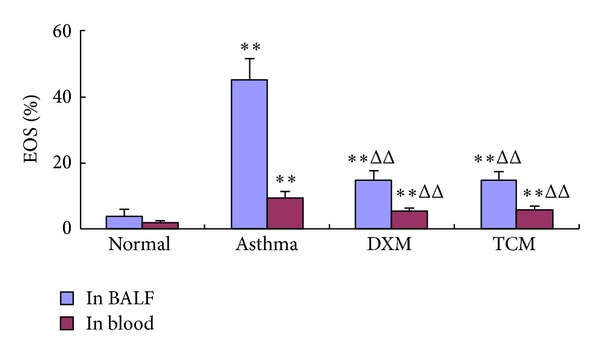
The percentages of EOS in BALF and blood.

As shown in Table 2, the total number of leukocytes in BALF of asthma group (17.77 ± 1.89) was significantly increased compared with normal group (8.44 ± 1.97, *P* < 0.01) and was significantly higher than those of TCM group (13.62 ± 2.18, *P* < 0.01) and DXM group (12.9 ± 1.57, *P* < 0.01). In addition, the percentage of Eos (Figure 2) in TCM group (14.49 ± 1.92) has no significant difference with DXM group (15.08 ± 2.60, *P* > 0.05) but was significantly lower than that of asthma group (45.05 ± 6.64, *P* < 0.01) and was higher than that of normal group (4.01 ± 1.93, *P* < 0.01). The percentages of neutrophils, epithelial cells, and lymphocytes were remarkably higher in the asthma group when compared with TCM group (*P* < 0.01) and DXM group (*P* < 0.01). As a positive control group, DXM group showed a similar suppressive effect on leukocyte influx into BALF, which indicated that TCM group (*P* < 0.01) was more favorable to treat with asthma.

### 3.2. Effects of TCM on OVA-Induced Total and Differential Leukocytes in Blood

**Figure 3:**
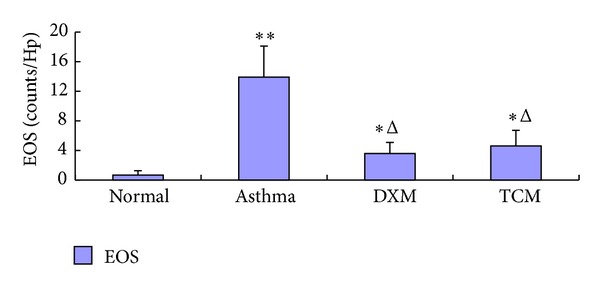
The changes of EOS infiltrating in the lung tissue.

As shown in Table 3, compared with the normal group (10.03 ± 2.74), a total number of leukocytes in blood were significantly increased in asthma group (18.16 ± 2.27, *P* < 0.01) and were significantly higher than that of TCM group (13.40 ± 1.67, *P* < 0.05) and DXM group (13.13 ± 1.42, *P* < 0.05). In addition, the percentage of Eos (Figure 3) in TCM group (5.93 ± 1.12) has no significant difference with DXM group (5.43 ± 1.12, *P* > 0.05) but was significantly lower than that of asthma group (9.70 ± 1.86, *P* < 0.01) and was higher than that of normal group (1.97 ± 0.79, *P* < 0.01). The percentages of neutrophils, epithelial cells, and lymphocytes were remarkably higher in the asthma group when compared with TCM group (*P* < 0.01) and DXM group (*P* < 0.01). As a positive control group, DXM showed a similar suppressive effect on leukocyte influx into BALF, which indicated that TCM group (*P* < 0.01) was more favorable to treat with asthma.

### 3.3. Effects of TCM on OVA-Induced EOS Airway Inflammation

A large number of Eos infiltrations can be seen through the lung tissue of asthma group (13.9 ±4.21, *P* < 0.01), which were significantly increased compared with the normal group (0.67 ± 0.59, *P* < 0.01) and were significantly higher than that of TCM group (4.6 ± 2.12, *P* < 0.01) and DXM group (3.6 ± 1.49, *P* < 0.01). There was no different from that of DXM group and TCM group (Figure 3).

### 3.4. The Levels of TNF-*α* and IL-4 in BALF and Serum

**Figure 4:**
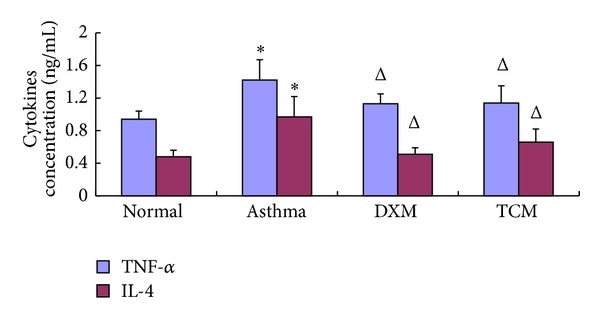
The concentration of TNF-*α* and IL-4 in serum.

**Figure 5:**
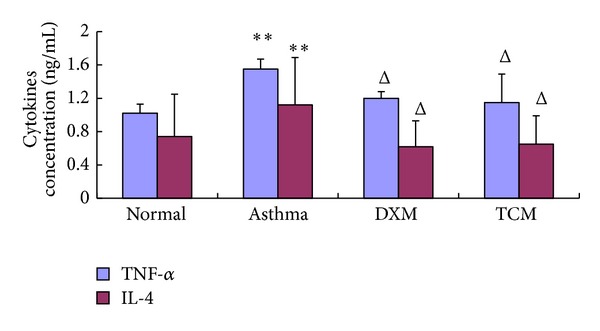
The concentration of TNF-*α* and IL-4 in BALF.

As shown in Figures 4 and 5, the OVA-induced asthma group significantly increased the concentrations of TNF-*α* and IL-4 in BALF in comparison to the normal group (*P* < 0.01, *P* < 0.01). Moreover, the concentrations of TNF-*α* and IL-4 in serum of the TCM group (1.14 ± 0.21, 0.66 ± 0.16) were obviously lower than the asthma group (*P* < 0.05, *P* < 0.01) and have no significant difference with DXM group (1.13 ± 0.12, 0.51 ± 0.08).

### 3.5. Effects of TCM on OVA-Induced EOS in Lung Tissue Biopsy

**Figure 6:**
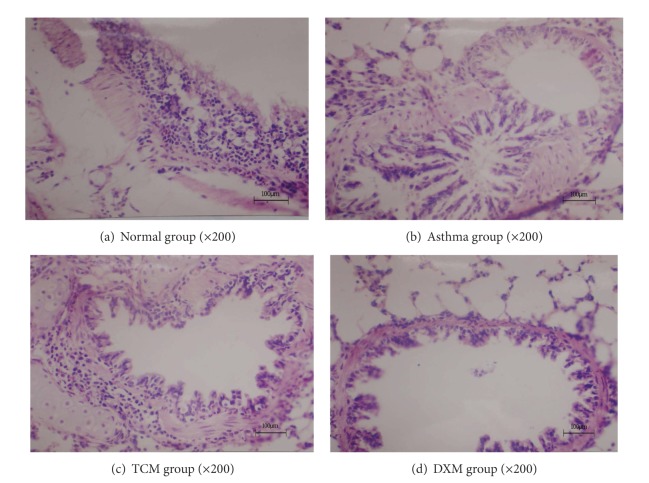
The airway inflammation in OVA-induced Guinea pigs. Lung tissue sections of OVA-induced/challenged Guinea pigs, stained with H&E and examined using light microscopy (magnification 200x). Scale bar = 100 *μ*m.

Lung tissue sections of OVA-induced Guinea pigs were stained with H&E and examined using light microscopy. Histological evaluation of the lung tissue demonstrated that, compared with the normal group, numerous inflammatory cells were observed in the lung interstitium around airways and blood vessels in the asthma group (Figure 6(b)). The results showed that only a few Eos and lymphocytes were distributed in the small bronchus of the normal group (Figure 6(a)). Guinea pigs treated by TCM significantly downregulated the accumulation of mucus in the airways and prevented the accumulation of Eos in BALF after OVA challenge (Figure 6(c)) compared with the asthma group. Likewise, DXM group showed a substantial reduction in inflammation and Eos infiltration into lungs (Figure 6(d)). Mucosal epithelium was tidy and the lung tissue was basically normal without obvious pathology change.

## 4. Discussion

OVA-induced asthma is a complicated inflammatory disease characterized by reversible airway obstruction, airway remodeling, airway inflammation, and AHR. The accompanying airways inflammation is characterized by infiltration of the airway wall with mast cells, lymphocytes, and Eos in the bronchial epithelium and lamina propriety [20, 30, 31]. In recent years, inhaled corticosteroids, leukotriene receptor antagonists, or *β*2-agonists are considered the most effective means of reducing airway inflammation, symptoms, and morbidity in patients with asthma. However, these treatments can produce potential negative side effects and do not consistently ameliorate airway inflammation in many asthmatic individuals [26, 32]. Thus, a safe and effective method is needed for the treatment of asthma.

The exact mechanism of herbs medicines on asthma is not entirely clear, but it is considered to be dependent on many persistent inflammatory cells, including infiltrating lymphocytes, Eos, basophils, and macrophages, and activated, and then synthesis and release of various pro-inflammatory mediators and cytokines [7, 20, 33, 34]. In this study, we have demonstrated a correlation between sihuangxiechai decoction and asthma. Dong et al. [27] showed evidences of lung tissue histological assay that various degrees of inflammation appeared in all OVA-induced animals. However, the asthma rats presented with more severe inflammation (inflammatory cell influx) than the treatment groups, and generally more serious inflammation was found in the DXM group than in TCM group. Our data showed that the total number of leukocytes in BALF and blood of asthma group was significantly higher than the other groups (*P* < 0.01), and rats treated with drugs showed substantial decrease compared with asthma rats (*P* < 0.01). This result suggested that sihuangxiechai decoction has an effect on the OVA-induced asthma. In addition, Regal [35] and Holgate et al. [36] concluded that Eos airway inflammation has been shown to be one of the basic features of allergic asthma. In our study, the percentage of Eos infiltrating in TCM group was significantly decreased in BALF and blood after OVA challenge (Figure 2) compared with the asthma group. Likewise, DXM group showed a substantial reduction in inflammation and eosinophilic infiltration into lungs (Figure 6(d)). These findings are consistent with the presented evidence for a significant increase in serum Eos level in TCM group.

Guan et al. [37] demonstrated that adequate assessment of inflammatory cells, cytokines, chemokines, and anti-inflammatory molecules is essential for understanding, monitoring, and treating lung diseases. Additionally, Stow et al. [38] concluded that IL-1*β*, IL-4, IL-13, and TNF-*α* have been found to reduce the severity of the inflammatory reaction. Larché et al. [39] and Bisset and Schmid-Grendelmeier [40] showed that allergic asthma is characterized by unbalance of Th1/Th2 cell and recruitment of type-2 T helper (Th2) cells. Th1 cells mainly excrete cytokines like IL-2, IFN-*α*, and TNF-*α*; Th2 cells mainly excrete cytokines like IL-4, IL-5, IL-6, IL-9, and IL-13 [27]. Through the manufacture and release of proinflammatory mediators, Eos can amplify the expression of Th1, Th2, and Th17 cytokines and chemokines suggesting that they play a vital role in the adaptive immune responses [41]. Activated Eos can secrete some basic proteins which may also be associated with the pathophysiology of asthma such as MBP, ECP, and EPO [8]. Previous studies have demonstrated that MBP can increase vascular permeability, bronchoconstriction, and airway epithelial damage; then remodeling is associated with more severe airflow obstruction, and AHR made in asthma. In our study, we found that the concentration of IL-4 and TNF-*α* in BALF or blood of TCM group both was markedly lower than that of asthma groups (*P* < 0.05, *P* < 0.01) and has no significant difference with DXM group (*P* < 0.05, *P* < 0.05). These results indicated that IL-4 and TNF-*α* were involved in the pathogenesis and development of asthma. Furthermore, all these results indicated that sihuangxiechai decoction had an inhibitory effect on the contents of IL-4 and TNF-*α*.

Sihuangxiechai decoction was composed of Xiaoqinglong decoction, Xiaochaihu decoction, and the powder of scorpion and centipede with the permit addition and subtraction. Huang et al. [42] concluded that Xiaoqinglong decoction and shegan mahuang decoction were widely used in clinic and received good curative effect. In this prescription,* Astragalus* with honey-fried herba ephedrae, stir-baked radix scutellariae, rhizoma polygonati, scorpio, centipede, and radix bupleuri have also been used as a traditional herb with sedative, antispasmodic, stomachic, tonic, diuretic, and topical bactericidal properties in order to enhance the effect of anti-inflammatory, antiallergic, antirheumatism, and immunosuppressive. In our study, inflammatory infiltration cells in or around the bronchus were significantly decreased in the TCM group more than in the asthma group, suggesting that sihuangxiechai decoction has curative effect on the bronchus inflammation of asthma (Figure 6).

In conclusion, this study clearly shows that sihuangxiechai decoction not only has evident antifebrile, antitussive, expectorant, and antiasthmatic effects but also relieves clinical symptoms and signs of asthma. Both of the TCM and DXM groups showed that Eos infiltration was reduced in various degrees and thus confirmed that the prescription can not only reduce Eos counts in peripheral blood but also decrease the possibility of EOS recruiting into airway. Additionally, prescriptions can suppress the damage of toxic proteins which were released by EOS and the formation of AHR. In summary, OVA-induced asthma treated by sihuangxiechai decoction is through multiple mechanisms to achieve their good treatment; at the same time, this method has provided the scientific basis for the application of clinical.

## 5. Conclusions

This study showed that sihuangxiechai decoction not only reduced the infiltration of leukocytes (especially Eos) but also suppressed histopathological changes such as airway remodeling and AHR. In conclusion, the results demonstrated that sihuangxiechai decoction might be a new potential therapy for the management of asthma in humans and for suppressing airway inflammation in a rat model of bronchial asthma. Further studies should be undertaken to clarify its detailed mechanism of action.

## Figures and Tables

**Table 1:** The main component of sihuangxiechai decoction.

Herbal medicine	Mass (g)
Astragalus	9
Honey-fried herba ephedrae	9
Stir-baked radix scutellariae	9
Rhizoma polygonati	9
Scorpio	6
Centipede	6
Radix bupleuri	9

**Table 2:** Counting and classification of leukocytes in BALF by treatment with TCM (%, $\overset{-}{x}\pm S$).

Classification	Normal group	Asthma group	DXM group	TCM group
Leukocyte	8.44 ± 1.97	17.77 ± 1.89**	12.90 ± 1.57**^ΔΔ^	13.62 ± 2.18**^ΔΔ^
Neutrophils	5.08 ± 2.62	12.34 ± 2.61**	9.31 ± 0.63**^Δ^	9.28 ± 0.98**^Δ^
Epithelial cells	4.32 ± 0.45	12.35 ± 1.44**	1.74 ± 0.40**^ΔΔ^	1.10 ± 0.41**^ΔΔ^
Eosinophilic	4.01 ± 1.93	45.05 ± 6.64**	15.08 ± 2.60**^ΔΔ^	14.69 ± 2.92**^ΔΔ^
Lymphocytes	3.90 ± 0.97	4.20 ± 0.51	2.60 ± 1.30*^Δ^	2.93 ± 0.43*^Δ^
Macrophage-monocytes	83.04 ± 3.01	25.42 ± 6.21**	72.00 ± 5.59**^ΔΔ^	73.62 ± 5.59**^ΔΔ^

*P < 0.05 and ∗∗*P* < 0.01 when compared with normal group; ^Δ^ *P* < 0.05 and ^ΔΔ^ *P* < 0.01 when compared with asthma group.

**Table 3:** Counting and classification of leukocytes in blood by treatment with TCM (%, $\overset{-}{x}\pm S$).

Groups	Normal group	Asthma group	DXM group	TCM group
Leukocytes	10.03 ± 2.74	18.16 ± 2.27**	13.13 ± 1.42*^Δ^	13.40 ± 1.67*^Δ^
Eosinophils	1.97 ± 0.79	9.70 ± 1.86**	5.43 ± 1.12**^ΔΔ^	5.93 ± 1.12**^ΔΔ^
Neutrophils	40.14 ± 8.13	62.66 ± 7.97**	50.67 ± 3.20*^ΔΔ^	53.33 ± 3.98^∗∗Δ^
Lymphocytes	47.97 ± 4.06**	27.07 ± 1.98**	43.93 ± 1.57**^ΔΔ^	60.25 ± 4.65**^ΔΔ^

*P < 0.05 and **P < 0.01 when compared with normal group; ^Δ^ *P* < 0.05 and ^ΔΔ^ *P* < 0.01 when compared with asthma group.
